# Validating a Patient-Reported Comorbidity Measure with Respect to Quality of Life in End-Stage Renal Disease

**DOI:** 10.1371/journal.pone.0157506

**Published:** 2016-06-13

**Authors:** Maxi Robinski, Franz Strich, Wilfried Mau, Matthias Girndt

**Affiliations:** 1 Institute for Rehabilitation Medicine, Medical Faculty of the Martin Luther University Halle-Wittenberg, Halle (Saale), Germany; 2 Department of Internal Medicine II, Medical Faculty of the Martin Luther University Halle-Wittenberg, Halle (Saale), Germany; 3 Center for Health Sciences of the Martin Luther University Halle-Wittenberg, Halle (Saale), Germany; Sao Paulo State University, BRAZIL

## Abstract

**Purpose:**

Medical record-derived comorbidity measures such as the Charlson Comorbidity Index (CCI) do not predict functional limitations or quality of life (QoL) in the chronically ill. Although these shortcomings are known since the 1980s, they have been largely ignored by the international literature. Recently, QoL has received growing interest as an end-point of interventional trials in Nephrology. The aim of this study is to compare a patient-reported comorbidity measure and the CCI with respect to its validity regarding QoL.

**Methods:**

The German Self-Administered Comorbidity Questionnaire (SCQ-G) was completed by 780 adult end-stage renal disease-patients recruited from 55 dialysis units throughout Germany. Acceptance was evaluated via response rates. Content validity was examined by comparing the typical comorbidity pattern in dialysis patients and the pattern retrieved from our data. Convergent validity was assessed via kappa statistics. Data was compared to the CCI. Linear associations with QoL were examined (criterion validity).

**Results:**

The SCQ-G was very well accepted by dialysis patients of all ages (response rate: 99%). Content validity can be interpreted as high (corresponding comorbidity items: 73.7%). Convergent validity was rather weak (.27≤*ρ*≤.29) but increased when comparing only concordant items (.39≤*ρ*≤.43). With respect to criterion validity, the SCQ-G performed better than the CCI regarding the correlation with QoL (e.g., SF-12-physical: SCQ-G total score: *ρ* = -.49 vs. CCI: *ρ* = -.36).

**Conclusions:**

The patient-reported measure proved to be more valid than the external assessment when aiming at insights on QoL. Due to the inclusion of subjective limitations, the SCQ-G is more substantial with respect to patient-centered outcomes and might be used as additional measure in clinical trials.

## Introduction

Comorbidity is a decisive factor in several clinical outcomes, such as mortality [1,2], the duration of in-patient stays, or quality of life (QoL) with chronic diseases [3,4]. Especially in end-stage renal disease (ESRD), comorbidity is an important determinant for dialysis treatment success and QoL [5,6] because ESRD-patients’ comorbidity covariates with the choice of a specific dialysis treatment option [7,8,9]. Young and less comorbid patients, for example, are assigned to peritoneal dialysis (PD) rather than hemodialysis (HD). In contrast, older patients who suffer from ESRD plus high comorbidity are more likely treated with HD. Since the choice of the dialysis treatment modality strongly influences the life of patients due to different extents of patient autonomy [10], it is important to accurately quantify comorbidity and its related burden in the chronically ill [11]. Per definition, comorbidity manifests in the occurrence of one or more diseases with the existence of one indexed disease [4]. With reference to current research in chronic kidney disease (CKD) as *the* indexed disease, hypertension, diabetes and heart failure typically co-occur as concordant comorbid diseases (causing CKD); whereas asthma, cancer or rheumatoid arthritis typically co-occur as discordant comorbid diseases (accompanying CKD) [12,13].

### Externally assessed comorbidity

To quantify comorbidity, information is obtained from medical records; diagnoses are usually weighted and summed. This method may be described as *externally assessed* comorbidity, for which many instruments exist [4,14–17]. In the international literature, the Charlson Comorbidity Index (CCI) [18] ranks as the most established one for different purposes, even though it was originally designed as a risk measure of mortality attributable to comorbidity. However, the calculation of a complete index by externally administered scoring lists is not always validly possible, since medical records are often incomplete [5]. Moreover, it needs extensively trained professionals for the correct indexing, as is true for the CCI. In fact, without training to warrant interrater-reliability, the accuracy between assessments of medical staff has been shown to be only moderate (e.g., for comorbidity [14]; for dementia [19]; for intensive care patients [20]). Besides, externally assessed comorbidity scores fail to reveal any information regarding functional limitations in patients’ everyday life or subjectively perceived consequences of comorbid diseases. To obtain insights into patients’ QoL and the impact of comorbidity, solely “counting” their diseases may not lead to adequate conclusions. For example, the subjective appraisal plays an important role on the effect of perceived stress due to chronic diseases [21,22]. Hence, it is very likely that externally assessed and self-reported comorbidity differ to some extent and that the patients’ statement contains valuable hints regarding actual QoL. Although the aforementioned shortcomings of external comorbidity measures are known since the 1980s, they have received little attention in the literature so far.

### Patient-reported comorbidity

One instrument to measure subjectively perceived comorbidity is the Self-Administered Comorbidity Questionnaire (SCQ) proposed by Sangha and colleagues [3]. Its German version (SCQ-G) shows good consensus and test criteria [23]. Convergent validity regarding the CCI in non-ESRD-samples is satisfying (*r* = .32 to .55 for the English version [3], *r* = .48 to .49 for the German version [23]). Furthermore, the SCQ proves prognostic validity regarding health-related QoL after one year [3], in-patient stays, and the self-reported treatment success [23]. Within a multivariate approach by Stolwijk et al. [24], the SCQ and a modified version contributed independently to physical function, health-related QoL and work disability in an ankylosing spondylitis sample. Finally, the SCQ outclasses the CCI in situations when medical reports are incomplete or missing, it saves human resources and offers an economic measure of comorbidity especially in large samples. In contrast to the CCI, the SCQ may be less adequate to predict mortality, since patients tend to over-report health-related problems [23]. In addition, items in diverse comorbidity survey instruments often differ regarding the recorded diseases and body systems. This also applies for the SCQ vs. CCI, and these aspects might negatively affect the cross-validity of a patient-administered instrument compared to an external comorbidity assessment.

### Study aims and research questions

Until now, the SCQ has not been validated in ESRD-patients. Furthermore, little is known about the question whether it would be a more adequate measure of comorbidity with respect to QoL than an external assessment. Hence, the present study aims to validate the SCQ in ESRD-patients and link this instrument to the patient-centered outcome “quality of life”. We will present a cross-sectional approach and refer to well-established test quality criteria.

#### Acceptance

How well do dialysis patients understand the items of the SCQ-G and know what to do within the survey?

#### Validity

How well does the SCQ-G cover typical comorbid diseases in a dialysis patient sample (content validity)?

- How does the SCQ-G relate to externally assessed comorbidity in ESRD-patients (convergent validity)?
- How does the SCQ-G relate to health-related QoL in ESRD-patients (criterion validity)?

## Methods

### Study design and sampling

The study was carried out within the framework of the CORETH-project (funded by the German Federal Ministry of Education and Research), a multicenter observational survey registered in the German Clinical Trials Register (#DRKS00006350) [25]. The project addresses the decision for either HD or PD. Hence, PD patients were enrolled at a much higher rate than suggested by the use of this treatment among German dialysis patients. Detailed information about the study design has already been published [10,25,26]. Patients were recruited from May 2014 to May 2015 from 55 dialysis units all over Germany. Local nephrologists screened the patients; two trained study nurses obtained informed written consent and surveyed the patients using standardized questionnaires. The time of ESRD-patients’ study entry was set at 6 to 24 months after initiation of dialysis. The time criterion ensured the absence of any acute complications or adaptation problems during the very early phase of dialysis. Moreover, inclusion criteria (absence of acute psychiatric symptoms, ability to read and understand the questionnaire, ability to provide written consent, age 18 years or older) ensured that patients were able to self-rate their comorbidity and functional limitations.

### Outcome measures

#### Externally assessed comorbidity

The Charlson Comorbidity Index (CCI) [18] is a 19 item-derived score to estimate the mortality of patients with several comorbid diseases. The CCI score strongly depends on the patients’ age, since each decade above 40 years adds an extra point to the CCI total score. Without considering age, the CCI score ranges from 0 to 37. The instrument displays a good test-retest-reliability (*r* = .92 [27]) and shows good cross-validity (Davies score: *r* = .80 [14]).

#### Patient-reported comorbidity

The German Self-Administered Comorbidity Questionnaire (SCQ-G [23]) addresses 13 body systems with three binary questions regarding the occurrence of health-related problems (*problem score*), received treatment or medication (*treatment score*), and experienced limitations in everyday life (*limitation score*). The *total score* of the SCQ-G is calculated as the sum of the affirmed items from all three sub-scores. The SCQ-G total score ranges from 0 to 39. The original version of the SCQ further contains three open items where participants can specify additional diseases (maximum score in this case = 48). The qualitative analysis of these open answers can be illuminative for the single case-evaluation of comorbidity. However, we excluded these items within the present study as did Sangha et al. [3], since they did not add value to quantitatively validate the SCQ. So far, no official test manual for the SCQ has been published. Information on convergent validity with respect to the CCI is based on a German sample of total endoprothesis-patients (*N* = 218 [23]).

#### Health-related QoL

The German version of the Short Form Health Survey (SF-12 [28]) assesses health related QoL from the patient perspective. The SF-12 is the short version of the SF-36 and contains the two dimensions *mental* (SF-12-MSC) and *physical* health (SF-12-PSC). The scores can range from 0 to 100 with *M* = 50 (*SD* = 10). Higher scores indicate a higher health-related QoL. Internal consistency (.70≤*Cronbach’s α*≤.87) and validity are widely satisfactory.

### Statistical analyses

#### Acceptance

The participants’ response behavior was examined to explore understandability and acceptance of the SCQ-G. For this, general response rates were inspected for all three SCQ-G-scores. The acceptance of the SCQ-G can be interpreted as high if every item on every score is answered. Following the instruction of the SCQ-G, patients should only answer items on the treatment and limitation score if the respective health-related problem had been previously marked with “yes”. For problems marked with “no”, the limitation and treatment scores should be skipped (indicating “0-0-0”).

#### Validity

**Fig 1:**
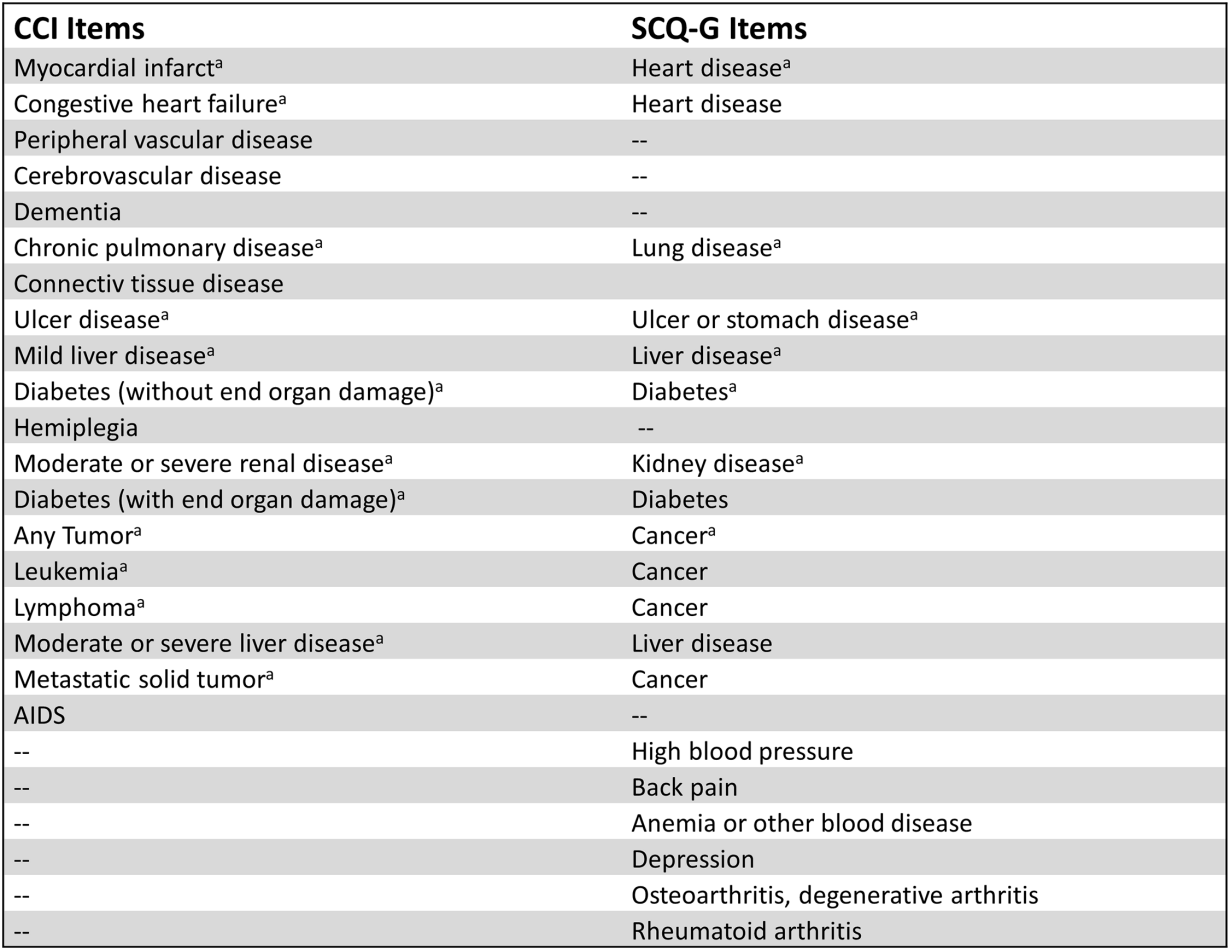
Convergence of the CCI and the SCQ-G. (Note: CCI = Charlson Comorbidity Index, SCQ-G = Self-Administered Comorbidity Questionnaire-German version, ^a^Items included in the adapted versions respectively).

Within the present approach *content validity* is defined as the extent to which items of the SCQ-G correspond with comorbid diseases that typically (co-)occur in CKD-patients [13]. In case of content-valid comparability between the SCQ-G items and the typical comorbidity pattern, the frequency of the respective patient-reported comorbid diseases should be analogous to previous findings [13]. *Convergent validity* refers to the correspondence of at least two instruments which are theoretically related (and expected to measure one concept). Assuming the CCI to be a well-established standard, the convergent validity of the SCQ-G was estimated by comparing SCQ-G and CCI scores. Correlation coefficients were computed for the original CCI and SCQ-G scores as well as the adapted versions containing only convergent items (see Fig 1). For comorbid diseases that were equally addressed by the SCQ-G and the CCI, kappa statistics have been estimated. Kappa represents the extent of matching between two sources of assessment with reference to a comorbid disease. In other words, kappa focuses the degree of concordance between the CCI as an external source and the SCQ as a self-assessment. The advantage of Kappa compared with percentage agreement is the chance correction. Due to the pre-selected sample of ESRD-patients it should be noted that substantial imbalances for the marginal distributions were to be expected for some comorbid diseases (e.g. kidney disease) and kappa statistics could be insufficient indices of concordance [29,30]. Since the chance-correcting component of kappa depends on the true prevalence, low kappa values can occur despite high empirical agreement [31]. To overcome this restriction, the *overall agreement* as well as the *proportions of specific agreement* between CCI and SCQ-G was additionally assessed. The overall agreement is defined as the ratio of total accordance between CCI and the SCQ-G for both, positive and negative judgements divided by the total number of cases [3]. The proportions of specific agreement are analogous to sensitivity and specificity but do not have a reciprocal relation [29,30]. Finally, *criterion validity* is defined as the relation between the investigated concept and an external criterion measure. For both SCQ-G and CCI, correlations with the two sum-scales of the SF-12 were assessed. All coefficients were tested for statistical significance using Fisher’s z-transformation [32]. Error probability was set to *α*≤.05.

### Ethical considerations

The study was carried out in accordance with the Code of Ethics of the Declaration of Helsinki and approved by the leading Ethics Committee of the University of Halle-Wittenberg, Germany. The Ethics Committees at every study site also approved the study protocol. Data safety in accordance with GCP-regulations has been guaranteed by the "Coordination Center for Clinical Studies Halle" (KKSH).

## Results

### Sample characteristics and descriptive statistics

The total sample consisted of 780 dialysis patients. The average age was 63.2 (*SD* = 15.1) with 32.6% of the patients being female. Further sample characteristics are presented in Table 1.

**Table 1:** Sample characteristics.

Characteristic	Total sample (*N* = 780)
Mean age (*SD*)	63.2 (15.1)
Gender, %	
Female	32.6
Male	67.4
Dialysis treatment modality, *n* (%)	
Hemodialysis	529 (67.8)
Peritoneal dialysis	251 (32.2)
Mean CCI (*SD*)	5.6 (2.3)
Mean SCQ-G problem score (*SD*)	4.0 (1.8)
Mean SCQ-G treatment score (*SD*)	3.3 (1.6)
Mean SCQ-G limitation score (*SD*)	2.1 (1.8)
Mean SCQ-G total score (*SD*)	9.3 (4.8)
Mean SF-12-MSC (*SD*)	51.7 (9.9)
Mean SF-12-PSC (*SD*)	37.4 (10.6)

**Note:** CCI = Charlson Comorbidity Index, SCQ-G = Self-Administered Comorbidity Questionnaire-German version, SF-12-MSC = Mental Sum Scale, SF-12-PSC = Physical Sum Scale.

### Validation results

#### Acceptance

On average, 99% of the patients indicated either “yes” or “no” for all items on the SCQ-G problem score. Less than 0.05% of the participants did not answer items on the limitation score or treatment score, even if they had previously marked the respective problem with “yes”. Furthermore, response patterns for diseases with treatment but without “yes” on the problem or limitation score (“0-1-0”) were observable for less than 0.05% of the participants. The same was true for patterns indicating patients to be limited by the respective comorbidity but not to have the problem and not to be under treatment (“0-0-1”). Hence, the acceptance and understandability of the SCQ-G can be interpreted as high.

#### Validity

The SCQ-G contained 73.7% of 19 substantial comorbid diseases which typically co-occur and have a greater population attributable risk (PAR) for CKD-patients [13]. Except alcohol misuse and peripheral vascular disease, all comorbid conditions that are not included in the SCQ-G refer to neurologic or psychiatric diseases, such as stroke/TIA, dementia or schizophrenia. Comparing frequency distributions, high blood pressure was mutually identified as the predominant comorbidity (Table 2). Consequently, content validity of the SCQ-G in ESRD-patients can be interpreted as good.

**Table 2:** Content validity analysis of the SCQ-G in kidney disease patients.

Content validity of SCQ-G items, %	SCQ-G problem score (*N* = 780)	Tonelli et al. [13] (*N* = 530,771)^a^
Heart disease	34.2	18.4
High blood pressure	69.3	46.6
Lung disease	15.3	17.4
Diabetes	33.2	17.8
Peptic ulcer disease	23.0	5.3
Liver disease	4.4	0.4
Blood disease	30.7	—
Cancer	6.7	5.9
Depression	11.1	11.3
Osteoarthritis	25.6	—^a^
Back pain	45.4	10.6
Rheumatoid arthritis	7.0	2.7

**Note:** SCQ-G = Self-Administered Comorbidity Questionnaire-German version.

^a^Alcohol misuse, peripheral vascular disease and neurological diseases added up to 32.6% at Tonelli et al. [13], but both conditions were not covered by the SCQ-G. In reverse, Tonelli’s analysis did not cover osteoarthritis.

Analyzing *convergent validity*, Spearman coefficients were low for the original CCI and SCQ-G-problem-score (*ρ* = .29), as well as the SCQ-G-total-score (*ρ* = .27). For the adapted versions, coefficients between the CCI and the SCQ-G-problem-score (*ρ* = .43) as well as the SCQ-G-total-score (*ρ* = .39) were slightly higher and therefore moderate [32]. All coefficients were significant at the *p* < .01 level. In order to investigate the agreement between higher CCI values and higher self-assessment values we only selected cases with mean values + 1 standard deviation in both indices. Doing so, n = 36 highly comorbid patients remained. Even in this sample, Spearman coefficients kept low (CCI and SCQ-G-problem-score [*ρ* = .28]; CCI and SCQ-G-total-score [*ρ* = .16]) and did not reach statistical significance.

Table 3 shows the agreement for those comorbid diseases mutually addressed by the SCQ-G and the CCI. Results from Sangha et al. [3] and Streibelt et al. [23] are presented for comparison. Sangha et al. calculated kappa statistics for individual items assessed with the original SCQ and the CCI. Streibelt et al. used kappa to estimate the “matching” between the SCQ German version and the CCI. It should be noted that these kappa values come from two different studies with two different populations (Sangha et al.: patients from general surgical care units; Streibelt et al.: orthopedic patients). Therefore, they merely provide an orientation in the context of existing empirical data. The *kappa statistics* retrieved from the present study ranged from fair agreement for heart disease, lung disease and cancer, to moderate agreement for liver disease, and to almost perfect agreement for diabetes (interpretation according to [33]). The agreement for peptic ulcer disease did not reach significance. The *overall agreement* exceeded 80% except for heart disease and peptic ulcer disease. *Positive agreement* was lowest for peptic ulcer disease, indicating little to no agreement regarding a positive diagnosis. However, *negative agreement* for peptic ulcer disease between the two scores was rather high. Inspecting differences in *prevalence*, the CCI was more restrictive for peptic ulcer disease, indicating patients to be more likely to experience this problem although there has been no medical record of it. Only slight differences were noted for the other diseases. In sum, considering only kappa statistics, rather fair to moderate agreement between the SCQ-G and the CCI was found. A more detailed inspection of the data in terms of positive and negative agreement as well as overall agreement indicated moderate to high coefficients between SCQ-G and CCI.

**Table 3:** Convergent validity analysis of CCI and SCQ-G problem score at the concordant item-level^a^.

Concordance (*N* = 780)	*Κ*	*Κ*^b^	*Κ*^c^	*P*_*o*_^d^, %	P_pos_^e^, %	P_neg_^f^, %	*P*_*o*_^c^^d^, %	*P*_SCQ-G_/*P*_CCI_^g^
Heart disease	.29**	.38**	.50**	70	.50	.78	78	.34/.27
Lung disease	.34**	.57**	.27**	85	.42	.91	88	.15/.11
Peptic ulcer disease	.01	.16**	.46**	75	.06	.85	92	.23/.03
Liver disease	.42**	.35**	.93**	95	.44	.97	99	.04/.04
Kidney disease	^h^	.52**	.79**	95	.97	^h^	98	.95/1.0
Diabetes	.84**	.83**	.90**	93	.89	.95	97	.33/.34
Cancer	.39**	.41**	.68**	90	.44	.95	90	.07/.10

**Note:** Kappa should be interpreted as follows: ≤0: no agreement; .01–.20: none to slight agreement, .21–.40: fair agreement, .41–.60 moderate agreement, .61–.80 substantial agreement, and .81–1.00 almost perfect agreement [33]. Important to note that when kappa values are moderate or lower, the confidence intervals around the obtained coefficients are sufficiently wide [34] and can hint that about half the data may be incorrect.

**p < .01;

^a^Data limited to items covered by both instruments;

^b^Data for comparison, retrieved from [21];

^c^Data for comparison, retrieved from [2];

^d^Proportion of overall agreement;

^e^Proportion of positive agreement;

^f^Proportion of negative agreement;

^g^Prevalence of the SCQ-G and the CCI;

^h^Data not calculated due to imbalanced marginal distribution.

*Criterion validity* analysis (Table 4) yielded low to moderate negative associations between the two sum-scales of the SF-12 and both comorbidity instruments, indicating a decreasing QoL with an increasing number of comorbid diseases. At that, coefficients for the SF-12-PSC were higher than for the SF-12-MSC. The correlation between the CCI and the SF-12-MSC did not reach significance, indicating no linear relation between externally assessed comorbidity and subjective mental well-being. In contrast, all scores derived from SCQ-G correlated significantly negatively with both QoL-aspects. Correlation coefficients between the CCI and the SCQ-G total score differed significantly for the SF-12-PSC (*z* = 3.46, *p* < .01). Coefficients were highest for the two SF-12 measures and the SCQ-G limitation score. Results indicate that perceived limitations due to comorbid diseases have a greater impact on QoL than the mere existence of comorbid diseases.

**Table 4:** Criterion validity analysis regarding QoL measures.

Criterion validity (*N* = 780), ρ	SF-12-MSC	SF-12-PSC
CCI	.06	-.36**
SCQ-G problem score	-.22**	-.45**
SCQ-G treatment score	-.15**	-.37**
SCQ-G limitation score	-.30**	-.52**
SCQ-G total score	-.25**	-.49**

Note:

** *p* < .01;

^*ρ*^ Spearman’s correlation coefficient;

CCI = Charlson Comorbidity Index; SCQ-G = Self-Administered Comorbidity Questionnaire-German version; SF-12-MSC = Mental Sum-Scale; SF-12-PSC = Physical Sum-Scale.

## Discussion

Well established instruments for comorbidity measurement such as the CCI show predictive value with respect to patients’ mortality, but do not contain any information about the subjective restrictions in everyday life. The aim of this study was to examine the SCQ-G in terms of test quality criteria and usability in an ESRD-sample. For this, the SCQ-G was compared to the CCI.

The overall response pattern reflects good acceptance and indicates the SCQ-G to be a comprehensive and economic instrument in a large sample. This finding underpins the previous suggestion [23] that the patient-report, compared to the CCI expert assessment, can save resources.

Regarding content validity, the items of the SCQ-G validly represent comorbidities typical for renal patients. Differences in frequencies between the patterns identified by Tonelli et al. [13] and the SCQ-G might occur due to different stages of kidney disease in the samples (CKD at Tonelli et al. vs. ESRD in the present study). However, since the cited analysis [13] is a registry study and our data are patient-reported, we may immanently expect some alterations in the incidence of comorbid diseases with a higher subjective appraisal-component, such as ulcer disease or back pain.

Associations between the original total scores of the SCQ-G and the CCI were low, but coefficients increased for the adapted versions. When comparing CCI and SCQ-G scores, several aspects have to be considered. Firstly, when comparing the adapted versions about one third of the items had to be left out. This may lead to some power reduction of the analysis. Secondly, the terminology of comorbid diseases strongly varies between both instruments. For example, while the CCI distinguishes between tumors without metastasis (exclude if > 5 y from diagnosis), leukemia, lymphoma and metastatic solid tumors, the SCQ-G only asks for cancer as an “overall” comorbid malignancy (Fig 1). Consequently, convergent validity statements should be derived carefully. Thirdly, the concordance between the SCQ-G and the CCI on the item-level strongly depends on the very item and used statistic: With few exceptions the kappa statistics yielded a lower concordance than was found in comparable studies with other patient samples. Streibelt et al. [23] found differences in *kappa statistics* to be highest for lung disease, heart disease and peptic ulcer disease. Data from Sangha et al. [3] showed the largest differences in kappa statistics for liver disease, cancer and peptic ulcer disease, whereas the *overall agreement* is comparable to our data. In sum, the matching between the patient-perception and the real existence of comorbidities is rather low and even lower than was found in a methodically related study [24], where kappa values ranged from .47 to 1.00. The low concordance may result from the fact that the patient-reported measure is weighted by limitations on daily activities. Hence, besides diseases themselves, it additionally considers their impact on the individual patient.

The relationships between the two comorbidity measures and the two aspects of QoL indicate a great advantage of the patient-centered assessment. The correlation to physical QoL was significantly higher for the SCQ-G than for the CCI. In contrast to the patient-reported comorbidity assessment, the external rating had no significant connection to mental QoL. Moreover, the advantage of the SCQ-G is not only to better relate to dialysis patients’ QoL, but to differentiate between the mere existence of a disease and its resulting limitation in daily life. Unsurprisingly, the strongest relation to the two QoL aspects was found for the SCQ-G limitation score. Hence, the SCQ-G is a more adequate measure than the CCI in the everyday clinical encounter, because it is more dedicated to patient-centered outcomes (such as QoL) than to externally rated clinical endpoints. Our findings strengthen the empirical results from Stolwijk et al. [24] indicating that the patient-assessment but not the CCI were correlated to patient-centered clinical outcomes. Since the SCQ facilitates conclusions about functional limitations that can impair (dialysis) patients’ autonomy, it may contribute to decisions about the optimal dialysis treatment modality [10]. We expect our 12 months-follow-up data by the end of this year. By testing patient-reported comorbidity as a predictor of other clinical outcomes, such as social functioning after one year, patients’ treatment satisfaction and quality-adjusted life years, we will further contribute to the question whether the SCQ is a valid and feasible measure of comorbidity [10,25].

### Limitations

Results should be generalized with caution due to the preselected sample of ESRD-patients. It should be borne in mind that due to these exclusion criteria patients with for example impaired sensory abilities (e.g., hearing loss) or proven dementia were precluded from the study. However, especially dementia is highly prevalent in ESRD-patients [35,36] and associated with higher comorbidity [37]. On the one hand, excluding these patients on the grounds of an impaired ability to self-assess their individual comorbidities can limit the representativeness of the sample and hence the external validity of findings. On the other hand, this might increase the internal validity of the study. Since we analyzed cross-sectional data, no measures of reliability were computed. Furthermore, the concept of comorbidity and hence the SCQ-G do not necessarily lead to a higher-level factor as it would be required for the calculation of internal consistency. In terms of future instrument development, it might be worthy to reconsider the idea of using one single comorbidity measure across cohorts with different indexed diseases [11]. Obviously, the CCI considers cancer more than does the SCQ. Additionally, the SCQ-G does not contain addiction, neurological or psychiatric conditions despite their relatively high incidence in CKD-patients [13]. In the clinical encounter this limitation can be overcome by means of analyzing the open items within the SCQ. However, unlike for the CCI, there is no statistical weighting of single comorbid diseases within the SCQ-G, nor is there any consideration of socio-demographic influences such as patients’ age. Regardless of this aspect, the SCQ-G weights comorbid diseases in another fashion than the CCI by including patients’ perception of limitations and is therefore “precise” in a patient-centered way.

## Conclusions

The SCQ-G proves to be a valid and feasible measure of comorbidity from the patient perspective and it shows relatively strong associations to different aspects of QoL. Due to the inclusion of patient-perceived limitations, the SCQ-G reveals more information with respect to medical counseling. Further research may improve its clinical application and interpretation.

## Compliance with Ethical Standards

### Ethical approval

All procedures performed in studies involving human participants were in accordance with the ethical standards of the institutional and/or national research committee and with the 1964 Helsinki declaration and its later amendments or comparable ethical standards.

### Informed consent

Informed consent was obtained from all individual participants included in the study.
